# Nursing management of a patient with calf muscle venous thrombosis complicated by bladder clot retention following oocyte retrieval: A case report

**DOI:** 10.1097/MD.0000000000048766

**Published:** 2026-05-15

**Authors:** Xuling Shen, Qun Wei, Aike Xu, Xinyi Yang, Xuechun Jiang, Meiling Xu, Zilian Wang

**Affiliations:** a Reproductive Center, Sir Run Run Shaw Hospital, Zhejiang University School of Medicine, Hangzhou, Zhejiang, China.

**Keywords:** bladder hemorrhage, bladder irrigation, calf muscle venous thrombosis, nursing care, oocyte retrieval

## Abstract

**Rationale:**

Venous thrombosis and hemorrhagic complications are rare but clinically significant risks during assisted reproductive treatments. This report describes comprehensive, evidence-based nursing interventions in a patient who developed calf muscle venous thrombosis and subsequent bladder hemorrhage with clot retention following oocyte retrieval.

**Patient concerns:**

A female patient undergoing controlled ovarian hyperstimulation presented with calf discomfort and later experienced gross hematuria and clot retention after transvaginal oocyte retrieval.

**Diagnoses:**

Intermuscular venous thrombosis of the calf with concurrent bladder hemorrhage.

**Interventions:**

A multidisciplinary, real-time nursing risk management strategy was implemented. Key interventions included enhancing the patient’s awareness of condition-related risks to promote active participation in care; continuous monitoring of bleeding risk through dynamic assessment of clinical indicators; strengthening acute-phase thrombosis management with early, evidence-based measures to prevent progression; optimizing bladder irrigation using a diluted hydrogen peroxide solution following careful evaluation of hemostatic and coagulation parameters; providing structured psychological support to address anxiety and emotional stress; and implementing phased, individualized follow-up nursing care after discharge, guided by Orem’s self-care deficit nursing theory.

**Outcomes:**

Bleeding was successfully controlled, and the patient achieved favorable postoperative recovery without complications. During follow-up, systematic nursing interventions and health education reinforced self-care and risk awareness. In February 2025, the patient underwent frozen embryo transfer, achieved a successful pregnancy and delivery.

**Lessons:**

This case highlights the importance of comprehensive, evidence-based nursing management to address rare but serious complications of assisted reproductive procedures. Integrating real-time risk monitoring, targeted hemostatic interventions, psychological support, and structured follow-up care can optimize outcomes, enhance patient self-management, and improve long-term reproductive success.

## 1. Introduction

Calf muscle venous thrombosis (CMVT) is defined as primary thrombosis confined to the gastrocnemius and soleus veins. It typically manifests with calf pain and lower-extremity edema, and in the absence of timely treatment, the thrombus may extend proximally to involve the deep venous system. This progression can lead to deep vein thrombosis and, in severe cases, pulmonary embolism (PE),^[1]^ a potentially fatal complication that requires urgent thrombolytic therapy. Transvaginal ultrasound-guided oocyte retrieval is a routine component of in vitro fertilization and embryo transfer in assisted reproductive technology. While transvaginal ultrasound-guided oocyte retrieval is generally regarded as safe, procedure-related complications, although uncommon, can be clinically significant. Reported complication rates include 0.5% to 8.6% for vaginal bleeding, approximately 0.021% for abdominal bleeding, 0.01% to 0.6% for pelvic infection, and 0.08% to 0.13% for ovarian torsion. In contrast, complications involving bladder injury are exceedingly rare, with only a few cases documented in the literature.^[2–4]^

We describe a complex case in which a patient with CMVT who had received thrombolytic therapy subsequently developed bladder hemorrhage following oocyte retrieval. This unusual perioperative presentation, characterized by the coexistence of active bleeding and a hypercoagulable state, markedly increased the difficulty and risk of clinical management.

In September 2024, the Reproductive Medicine Center of our hospital admitted a woman with a 3-year history of infertility secondary to polycystic ovary syndrome (PCOS) who was scheduled for assisted reproductive treatment. During controlled ovarian hyperstimulation (COH), she was diagnosed with CMVT and treated with anticoagulant therapy up to the day preceding oocyte retrieval. Postoperatively, she developed bladder hemorrhage complicated by clot retention and bladder distension. A multidisciplinary team (MDT) consultation was convened, and an individualized diagnostic and therapeutic plan was implemented in conjunction with targeted nursing interventions. The patient recovered without further complications.

This report highlights the clinical challenges and nursing strategies in managing rare but serious perioperative complications in assisted reproductive technology, providing insights that may inform risk assessment and individualized care in similar cases.

## 2. Clinical data

### 2.1. General information

A 30-year-old woman (body mass index 26.4 kg/m^2^), gravida 0, para 0, presented for in vitro fertilization and embryo transfer due to infertility attributed to PCOS. Her medical history was notable for abnormal liver function consistent with fatty liver disease and impaired folic acid metabolism. An oral glucose tolerance test confirmed the presence of insulin resistance.

**Figure 1.**
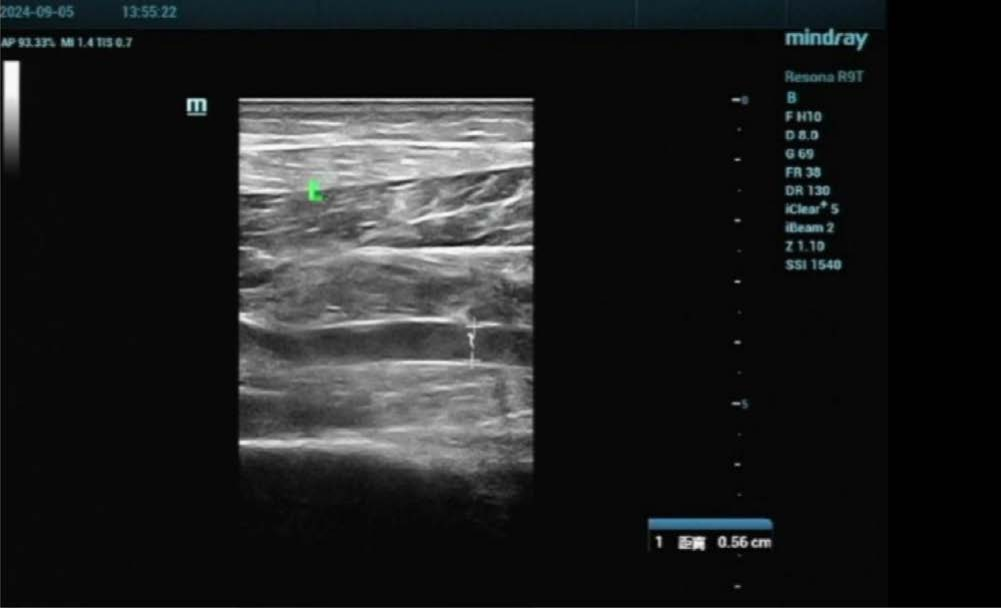
Doppler ultrasonography confirming thrombosis within the intermuscular vein of the left calf. AP = anteroposterior, DR = dynamic range, FR = frame rate, MI = mechanical index, SSI = spatial standard intensity, TIS = thermal index scan.

Following pretreatment with metformin and drospirenone/ethinyl estradiol, COH was initiated using a gonadotropin-releasing hormone antagonist protocol, with recombinant human follicle-stimulating hormone administered at a dose of 150 IU/day. On stimulation day 9, the patient developed swelling of the left calf and was subsequently diagnosed with intermuscular vein thrombosis (Fig. 1). Anticoagulation therapy with nadroparin calcium (0.4 mL/day, subcutaneously) was initiated and continued until the day preceding oocyte retrieval. Oocyte retrieval was performed 36 hours after dual triggering with human chorionic gonadotropin and triptorelin, resulting in the retrieval of 13 oocytes. Two hours after the procedure, the patient developed gross hematuria accompanied by clot formation.

### 2.2. Treatment process and outcome

**Figure 2.**
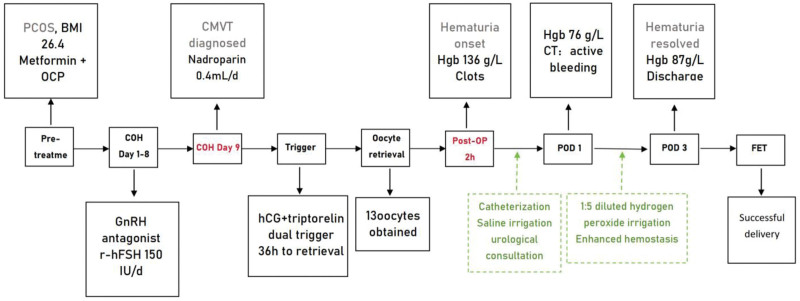
Timeline of clinical events. BMI = body mass index, CMVT = calf muscular venous thrombosis, COH = controlled ovarian hyperstimulation, CT = computed tomography, FET = frozen embryo transfer, GnRH = gonadotropin-releasing hormone, hCG = human chorionic gonadotropin, Hgb = hemoglobin, OCP = oral contraceptive pill, PCOS = polycystic ovary syndrome, POD = postoperative day, r-hFSH = recombinant human follicle-stimulating hormone.

**Figure 3.**
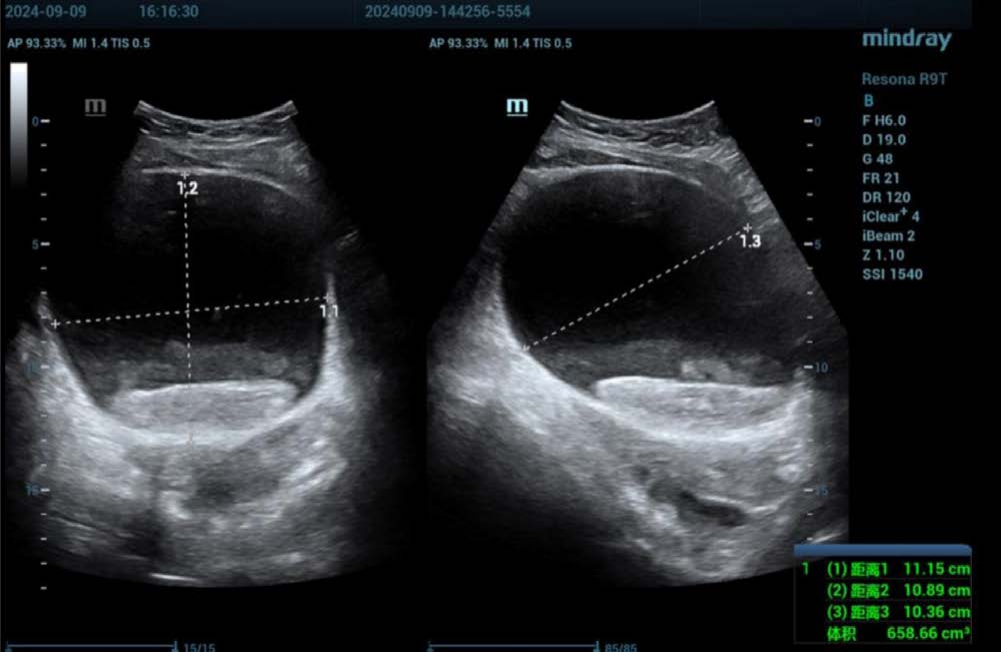
Ultrasonography demonstrating severe bladder distension. AP = anteroposterior, DR = dynamic range, FR = frame rate, MI = mechanical index, SSI = spatial standard intensity, TIS = thermal index scan.

**Figure 4.**
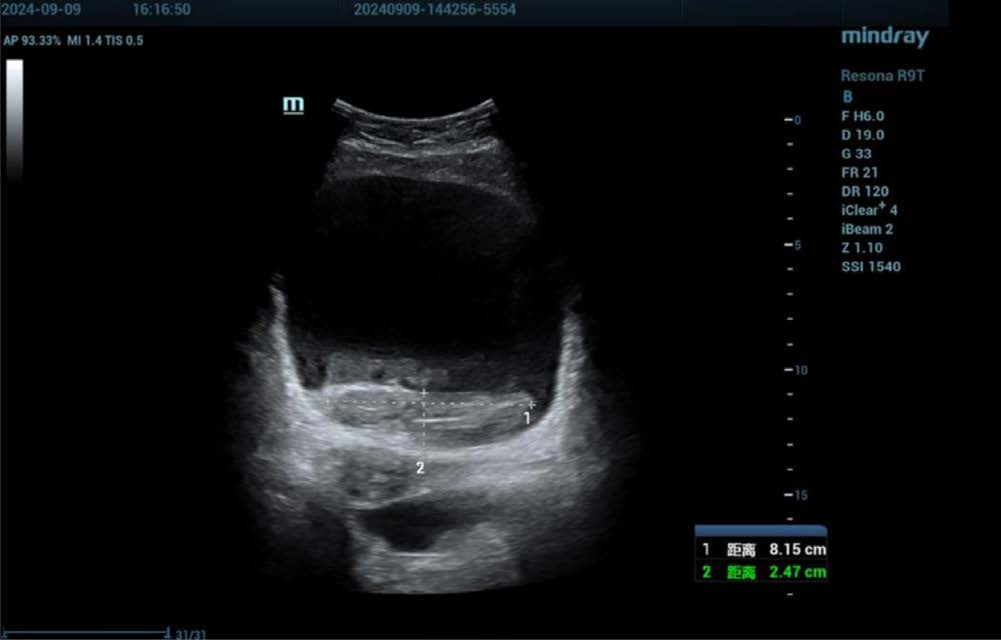
Ultrasonography revealing an 8.15 × 2.47 cm intravesical hematoma. AP = anteroposterior, DR = dynamic range, FR = frame rate, MI = mechanical index, SSI = spatial standard intensity, TIS = thermal index scan.

The initial hemoglobin concentration was 136 g/L. Within 1 hour of hematuria onset, the patient developed abdominal distension and dysuria. Ultrasonographic evaluation demonstrated marked bladder distension, with an 8.15 × 2.47 cm intravesical hypoechoic lesion without detectable blood flow (Figs. 2–4).

Following urologic consultation, indwelling urinary catheterization with continuous saline bladder irrigation was initiated. Despite the administration of antimicrobial and hemostatic therapies, the patient’s hemoglobin level declined to 76 g/L on postoperative day 1. The clinical course was further complicated by recurrent catheter obstruction secondary to blood clots and gas formation. Computed tomography imaging confirmed the presence of an intravesical hematoma with suspected ongoing bleeding. In response, bladder irrigation was transitioned to a 1:5 diluted hydrogen peroxide solution, and hemostatic therapy was intensified. Hydroxyethyl starch was administered for prevention of ovarian hyperstimulation syndrome (OHSS).

By postoperative day 3, hematuria had resolved, and the hemoglobin level stabilized at 87 g/L. Follow-up ultrasonography confirmed resolution of the intravesical clot. The urinary catheter was subsequently removed, and the patient was discharged in stable condition.

## 3. Nursing

### 3.1. Enhancing risk awareness and promoting active patient participation in care

The patient underwent 1 month of pretreatment with oral contraceptive pill prior to ovulation induction for the management of PCOS. At baseline, she exhibited multiple risk factors, including elevated body mass index, impaired hepatic function, and insulin resistance, which collectively placed her at increased risk for venous thromboembolism.^[5]^ During COH with gonadotropins, she developed synchronous growth of multiple ovarian follicles, accompanied by estradiol levels markedly exceeding physiologic ranges. This profile indicated an increased susceptibility to both OHSS and thromboembolic events. In anticipation of these risks, nursing staff identified her as high risk and implemented a personalized education program designed to prevent complications. Instruction was delivered through a combination of educational videos, illustrated handouts, and verbal counseling. The patient was advised to wear compression stockings, maintain adequate hydration, and engage in moderate physical activity. During stimulation, the patient recognized swelling of her left leg as abnormal and immobilized the limb as instructed before promptly returning to the clinic. Doppler ultrasonography confirmed CMVT. In accordance with medical orders, the nursing team administered daily subcutaneous low-molecular-weight heparin. Nurses collaborated with physicians to provide detailed guidance on monitoring thrombus progression and recognizing bleeding risks associated with anticoagulation therapy. The patient demonstrated a clear understanding of the instructions and actively adhered to the prescribed treatment and nursing plan.

### 3.2. Systematic evaluation of bleeding etiology and continuous dynamic monitoring

The patient had been diagnosed with CMVT prior to surgery and was treated with nadroparin calcium (0.4 mL subcutaneously once daily for 4 consecutive days) according to medical instructions. While clinically indicated, this regimen increased her risk of postoperative bleeding. Following oocyte retrieval, she developed 2 episodes of hematuria accompanied by clot passage. The nurse promptly informed the attending physician and coordinated a comprehensive diagnostic workup, including complete blood count, transvaginal color Doppler ultrasound of the uterus, adnexa, and pelvic cavity, and urinary system Doppler imaging. After MDT discussion, persistent hematuria with clot formation was determined to be most likely attributable to 2 primary factors: inadvertent bladder puncture during oocyte retrieval leading to mucosal or vascular injury and continued anticoagulation therapy with low-molecular-weight heparin for CMVT, which impaired coagulation function and manifested as hematuria.^[6]^

To mitigate the risk of hemorrhagic shock, targeted nursing interventions were implemented. Hemostasis and iron supplementation: Intravenous tranexamic acid was administered as prescribed, with careful monitoring for adverse reactions such as dizziness, nausea, vomiting, and visual disturbances; oral iron supplements were also initiated. Laboratory monitoring: On the first postoperative day, hemoglobin decreased to 76 g/L, and computed tomography suggested possible bleeding from the bladder wall; these findings were immediately reported, and blood was cross-matched in preparation for potential transfusion and cystoscopy. Early detection of shock: Vital signs were assessed every 30 minutes to promptly identify any signs of hemodynamic instability. Bladder decompression through positioning: The patient was maintained in bed with the head of the bed elevated 30 to 45 degrees to decrease bladder pressure and reduce bleeding risk. Patient education on hemorrhagic symptoms: The patient was counseled to promptly report signs such as fatigue, abdominal pain, gum bleeding, and melena to facilitate early recognition of complications. Infection prevention: Intravenous ceftriaxone was administered as prescribed, and strict catheter care protocols were followed, including regular disinfection of the catheter, timely emptying of the drainage bag, and daily replacement of the collection system.

### 3.3. Development of comprehensive, multi-objective intervention strategies with emphasis on acute-phase thrombosis management

An MDT was promptly convened, comprising specialists in reproductive medicine, reproductive nursing, urology, vascular surgery, diagnostic ultrasound, rehabilitation medicine, and mental health. The team conducted a comprehensive case review and formulated individualized diagnostic, therapeutic, and nursing strategies tailored to the patient’s evolving clinical status. The primary management priorities included the initiation of intravenous hemostatic therapy, minimization of invasive interventions whenever feasible, and close surveillance for potential proximal extension of the isolated distal venous thrombosis. The specific objectives of the intervention strategy were as follows: Promote venous return and prevent venous distension and stasis: The patient was instructed to wear graduated compression stockings. During management of bladder hemorrhage, intermittent elevation of the affected limb was encouraged to facilitate venous drainage. Maintain mobility and prevent thrombosis in the unaffected limb: Because continuous bladder irrigation limited the patient’s mobility and ability to reposition, nurses guided her in performing gentle in-bed exercises, including leg elevation and ankle pump exercises with the unaffected limb.^[7]^ In collaboration with the Department of Rehabilitation Medicine, neuromuscular electrical stimulation was applied to the unaffected calf to induce rhythmic muscle contractions, thereby activating the skeletal muscle pump, enhancing venous return, and reducing the risk of de novo thrombosis.^[8]^ Monitor closely for thrombotic progression and embolic complications: Meticulous surveillance of the known CMVT was undertaken to identify any signs of extension to deep vein thrombosis, PE, or post-thrombotic syndrome. Nursing staff adhered to strict monitoring protocols, measuring and documenting the maximum circumference of both lower extremities every 4 hours for serial comparison. Continuous assessments also included evaluation of skin color, temperature, and swelling in both legs. In addition, the patients were regularly screened for symptoms indicative of PE, including chest tightness, cough, pleuritic chest pain, and hemoptysis.

### 3.4. Evidence-based optimization of bladder irrigation protocols with gentle hemostasis and effective clot removal

D’Amico et al reported that the principal therapeutic approaches for bladder hemorrhage include bladder irrigation, cystoscopy-guided hemostasis, and arterial embolization therapy.^[9]^ In this case, bladder irrigation was prioritized following evidence-based nursing assessment and MDT consultation. This decision was based on several key considerations: the patient’s ongoing need for anticoagulation as a result of thrombophilia and CMVT, her strong desire to expedite recovery for subsequent assisted reproductive treatment, and the goal of optimizing patient satisfaction while minimizing invasive procedures such as cystoscopy.

In accordance with medical orders, a 22Fr 3-lumen double-balloon urinary catheter was placed to facilitate irrigation and prevent repeated catheter replacement, thereby reducing the risk of ureteral and bladder mucosal injury and minimizing complications such as infection and hemorrhage. On the day after oocyte retrieval, the patient developed complete bladder obstruction resulting from extensive clot retention. Conventional normal saline bladder irrigation was ineffective in clot evacuation. Subsequent MDT review identified several pathophysiologic mechanisms contributing to persistent intravesical bleeding: CMVT was associated with hyperfibrinolysis and hypofibrinogenemia, while preoperative administration of low-molecular-weight heparin further suppressed the coagulation cascade, impairing fibrin formation and resulting in fragile clots unable to maintain stable hemostasis.^[10]^ Gonadotropin-releasing hormone agonists and antagonists administered during ovulation induction may compromise vascular endothelial integrity, increase capillary fragility, and reduce vasoconstrictive capacity after hemorrhage, thereby limiting spontaneous hemostasis.^[11]^

Evidence from prior studies supports the use of hydrogen peroxide in this setting.^[12,13]^ Research has demonstrated that 3% hydrogen peroxide solution reduces clot adhesiveness by altering clot properties, facilitating their removal, while additional reports^[14,15]^ confirm that hydrogen peroxide enhances coagulation when applied to bleeding wounds. Based on these findings, the MDT recommended low-concentration hydrogen peroxide irrigation to simultaneously promote hemostasis and dissolve obstructing clots. The nursing team prepared an irrigation solution by diluting 3% hydrogen peroxide with 0.9% sodium chloride injection at a ratio of 1:5. To prevent hypothermia-related coagulopathy, the infusion bag was warmed in a water bath to 35 to 37°C before administration, as hypothermic irrigation fluid may impair platelet function, activate fibrinolysis, and exacerbate bleeding.^[16]^ Prior to irrigation, the catheter was clamped, and approximately 100 mL of the warmed solution was instilled intravesically at a rate of 50 to 60 drops per minute. The solution was retained for 3 to 5 minutes, after which the catheter was opened to allow drainage. This constituted 1 irrigation cycle, and the procedure was repeated 3 times. The irrigation effluent demonstrated a progressive color change from grossly bloody to light pink, with sequential evacuation of small clots. Throughout catheter clamping, the patient was closely monitored for signs of sympathetic hyperactivity – including abdominal distension, diaphoresis, tachycardia, or elevated blood pressure – which would have necessitated immediate discontinuation of the procedure. No adverse events occurred. Bedside ultrasound confirmed clearance of intravesical clots, after which continuous bladder irrigation with 0.9% sodium chloride was resumed to eliminate residual hydrogen peroxide. On the following day, repeat ultrasound confirmed the absence of recurrent clot formation. Hemoglobin levels improved to 87 g/L, reflecting clinical recovery compared with the nadir value. Per physician’s orders, the urinary catheter was removed, and spontaneous urination was subsequently achieved with clear urine output and no recurrence of hematuria.

### 3.5. Psychological intervention to enhance treatment compliance

During the course of bladder hemorrhage management, the patient exhibited marked anxiety and fluctuating adherence to treatment recommendations. Nursing staff identified these psychological changes during routine care and promptly reported their observations to the MDT. In response, a nurse-led psychological support initiative was established, providing multiple sessions of structured, in-depth counseling tailored to the patient’s immediate emotional distress. The primary objective was to identify and address psychosocial risk factors, stabilize the patient’s psychological state, and ultimately improve treatment compliance.^[17]^

Through repeated counseling sessions, the patient gradually disclosed her concerns, which centered on 4 key domains: comparison with peers undergoing concurrent ovulation induction who progressed without complications fostered a sense of “bad luck,” pessimism about treatment outcomes, and fear of eventual failure; prolonged bladder irrigation, enforced bed rest, and confinement to the hospital disrupted her circadian rhythm and produced diurnal inversion, which she described as feeling “like an experimental animal trapped in a cage,” resulting in profound isolation and loss of autonomy; uncertainty regarding the potential impact of hospitalization on subsequent embryo transfer and pregnancy success generated apprehension about long-term reproductive outcomes; and recurrent hematuria observed during bladder irrigation evoked dual anxieties – fear of uncontrolled hemorrhage and concern regarding progression of lower limb thrombosis – contributing to sustained psychological distress.

To mitigate these challenges, the nurse initiated direct communication with the patient’s spouse, providing education on psychological support strategies and fostering family participation in the care process. With informed consent from both the patient and her family, environmental modifications were also introduced: her bed was relocated from a corridor-adjacent location to a window-facing position, thereby restoring circadian rhythm cues through natural light exposure and improving well-being by facilitating visual connection with the external environment. Additionally, the MDT engaged the patient and her family in comprehensive discussions regarding treatment strategies and reproductive prognosis, addressing uncertainties and collaboratively formulating an individualized care pathway. Following these integrated interventions, the patient demonstrated marked improvement in mood, reduced anxiety, and notably enhanced adherence to treatment.

### 3.6. Development of a structured follow-up strategy and provision of continuous care

The patient, a young woman with PCOS, underwent transvaginal oocyte retrieval in which 15 follicles were aspirated and 13 oocytes successfully obtained. Owing to the number of retrieved oocytes, she was classified as being at high risk for OHSS. At the time of discharge, color Doppler ultrasonography revealed persistent thrombosis within the deep veins of the lower extremities. Following resolution of bladder hemorrhage, anticoagulation therapy was resumed as directed by the attending physician.

The importance of continued vigilance was underscored by existing evidence. Numerous studies^[18–20]^ have documented that patients with moderate-to-severe OHSS have an elevated risk of developing venous or arterial thrombotic events. Kret et al further reported that patients with isolated CMVT remain at high risk for recurrent venous thromboembolism.^[21]^ In such cases, appropriate anticoagulant therapy is effective in controlling acute venous thrombosis, and evidence suggests that extending treatment for 2 to 3 months after resolution markedly reduces recurrence rates.^[22]^ On the basis of these clinical considerations, and following detailed communication with the patient, nursing staff developed an individualized health education program and a structured follow-up strategy. The schedule consisted of weekly telephone follow-up during the first month after discharge, followed by biweekly calls until 6 months postpartum. The follow-up protocol targeted 5 key domains: Symptom monitoring: The patient was instructed to measure and compare the circumference of both lower legs daily, monitoring for pitting edema; if edema developed bilaterally or if asymmetric swelling with pain occurred, immediate hospital evaluation was advised. She was also instructed to monitor abdominal girth and body weight, with prompt medical consultation recommended for rapid weight gain or severe abdominal distension. Outpatient follow-up management: Per medical advice, Doppler ultrasonography of the lower extremities was scheduled every 2 weeks, alongside regular laboratory assessments including complete blood count and D-dimer, until venous recanalization was confirmed. At the 4-week visit, ultrasonography demonstrated restored patency of the left calf vein. Medication management: Post-discharge therapy included oral letrozole (5 mg once daily for 1 week) to reduce the risk of OHSS recurrence, combined with prophylactic low-molecular-weight heparin injections administered subcutaneously once daily. The patient was counseled to closely monitor for bleeding signs such as mucosal bleeding, petechiae, or gum bleeding. Dietary guidance: A high-protein, low-fat, easily digestible diet was recommended, supplemented with adequate dietary fiber to promote bowel motility and prevent constipation. Fluid intake was advised in divided doses, totaling 2000 to 2500 mL per day. Risk awareness and emergency preparedness: Targeted health education emphasized the necessity of continued anticoagulation therapy. Scenario-based teaching was provided to enhance the patient’s ability to recognize and respond to emergencies such as bleeding or thrombotic recurrence, thereby improving vigilance and self-management skills.

During hospitalization, the MDT also recommended genetic evaluation for thrombophilia. Post-discharge testing identified a plasminogen activator inhibitor-1 4G/4G genotype, confirming a high-risk profile. Prior studies^[23]^ have demonstrated that individuals with this genotype experience a hypercoagulable state associated with placental microvascular thrombosis, trophoblast dysfunction, and vascular remodeling, which significantly increases the risk of adverse pregnancy outcomes – including recurrent miscarriage, preeclampsia, intrauterine growth restriction, and stillbirth – relative to the general obstetric population. Furthermore, thrombus formation during pregnancy may interfere with the accuracy of prenatal screening markers, including pregnancy-associated plasma protein-A, beta subunit of human chorionic gonadotropin, and nuchal translucency measurements.

Accordingly, during the patient’s frozen embryo transfer cycle, nursing care was guided by Orem’s self-care deficit theory.^[24]^ A structured assessment of her capacity for self-care in the context of thrombophilia was conducted, identifying specific deficits. The nursing team provided comprehensive instruction on anticoagulant self-injection, recognition of potential complications, and psychological adaptation to the elevated pregnancy risks associated with thrombophilia. These interventions not only reduced anxiety arising from uncertainty but also significantly enhanced the patient’s self-efficacy, treatment adherence, and overall sense of control.

## 4. Discussion

The occurrence of CMVT in conjunction with bladder hemorrhage and clot retention following oocyte retrieval in patients undergoing ovulation induction represents an exceptionally rare but clinically significant complication. Such events not only compromise the patient’s treatment experience but, if inadequately managed, may negatively influence subsequent embryo transfer outcomes and thereby reduce the likelihood of achieving a successful clinical pregnancy. To minimize the risk of these complications, genetic testing may be considered in patients with a family history of documented abnormalities in folate metabolism. For individuals identified as high risk, initiation of prophylactic anticoagulation in strict accordance with established clinical guidelines and under physician supervision is warranted. In parallel, patient education must be strengthened to ensure awareness of thrombotic and hemorrhagic risks and to promote early recognition and timely reporting of warning symptoms.

In the perioperative management of patients with CMVT, predictive and preventive nursing strategies are essential. Preoperatively, careful evaluation of coagulation function should be conducted, and any abnormalities should be addressed before proceeding. Intraoperatively, particular vigilance should be directed toward minimizing the risk of vascular trauma by avoiding inadvertent puncture of adjacent tissues and organs. Postoperatively, meticulous monitoring is required to detect even subtle signs of bleeding, with prompt intervention to prevent progression to severe hemorrhagic complications. For patients at high risk of thrombosis, longitudinal follow-up must be intensified across the reproductive continuum – before conception, throughout pregnancy, and into the postpartum period. Enhanced surveillance enables early detection of thrombotic or hemorrhagic events, while targeted education equips patients to anticipate and manage potential complications. Particular attention should also be directed toward the possibility of aberrant results in prenatal testing that may arise from underlying hypercoagulable states. Through personalized, anticipatory education and comprehensive nursing care, risks can be minimized and patient safety optimized.

## Acknowledgments

We thank Phoebe Chi, MD, from Liwen Bianji (Edanz; www.liwenbianji.cn), for editing a draft of this manuscript.

## Author contributions

**Conceptualization:** Qun Wei.

**Methodology:** Xuling Shen, Aike Xu, Xinyi Yang, Xuechun Jiang, Meiling Xu.

**Formal analysis:** Xuling Shen.

**Investigation:** Xuling Shen.

**Supervision:** Zilian Wang.

**Project administration:** Xuling Shen, Qun Wei.

**Writing – original draft:** Xuling Shen.

**Writing – review & editing:** Xuling Shen, Zilian Wang, Qun Wei, Aike Xu, Xinyi Yang.
